# Posterior Reversible Encephalopathy Syndrome and Reversible Cerebral Vasoconstriction Syndrome: Clinical and Radiological Considerations

**DOI:** 10.3389/fneur.2020.00034

**Published:** 2020-02-14

**Authors:** Fabio Pilato, Marisa Distefano, Rosalinda Calandrelli

**Affiliations:** ^1^Fondazione Policlinico Universitario A. Gemelli – IRCCS, Rome, Italy; ^2^UOC Neurologia, Dipartimento di Scienze dell'invecchiamento, Neurologiche, Ortopediche e Della Testa-Collo, Rome, Italy; ^3^UOC Neurologia e UTN, Ospedale Belcolle, Viterbo, Italy; ^4^UOC Radiologia e Neuroradiologia, Dipartimento di Diagnostica per Immagini, Radioterapia Oncologica ed Ematologia, Rome, Italy

**Keywords:** reversible cerebral vasoconstriction syndrome, posterior reversible encephalopathy syndrome, RCVS, PRES, call-fleming syndrome, reversible posterior leukoencephalopathy syndrome, magnetic resonance imaging

## Abstract

Posterior reversible encephalopathy syndrome (PRES) and reversible cerebral vasoconstriction syndrome (RCVS) are relatively uncommon neurological disorders, but their detection has been increasing mainly due to clinical awareness and spreading of magnetic resonance imaging (MRI). Because these syndromes share some common clinical and radiologic features and occasionally occur in the same patient, misdiagnosis may occur. PRES is characterized by varied neurological symptoms including headache, impaired visual acuity or visual field deficit, confusion, disorders of consciousness, seizures, and motor deficits often associated to peculiar neuroradiological pattern even if uncommon localization and ischemic or hemorrhagic lesions were described. RCVS is a group of diseases typically associated with severe headaches and reversible segmental vasoconstriction of cerebral arteries, often complicated by ischemic or hemorrhagic stroke. Pathophysiological basis of PRES and RCVS are still debated but, because they share some risk factors and clinical features, a possible common origin has been supposed. Clinical course is usually self-limiting, but prognosis may fluctuate from complete recovery to death due to complications of ischemic stroke or intracranial hemorrhage. Neuroradiological techniques such as digital angiography and MRI are helpful in the diagnostic pathway and a possible prognostic role of MRI has been suggested. This review will serve to summarize clinical, neuroradiological features and controversies underlying both syndromes that may mislead the diagnostic pathway and their possible relationship with pathophysiology, clinical course, and prognosis.

## Introduction

Reversible cerebral vasoconstriction syndrome (RCVS) and posterior reversible encephalopathy syndrome (PRES), although relatively uncommon neurological disorders, have become increasingly recognized, mainly due to the spreading of brain magnetic resonance (MRI) and clinical awareness.

PRES, also called reversible posterior leukoencephalopathy syndrome, hyper-perfusion encephalopathy, or brain capillary leak syndrome, is an acute or subacute neurological disorder; even if each label describes a particular feature of the syndrome, none of them is completely satisfactory. Since the first systematic description by Hinchey et al. (1), risk factors of PRES including immunosuppression, malignancy, pre-eclampsia, renal failure, autoimmune disorders, sepsis, hypertension, transplantation, and chemotherapeutic medications remained unchanged even if it may occur also in healthy subjects.

RCVS, previously named isolated benign cerebral vasculitis, Call or Call-Fleming syndrome, and migrainous vasospasm are a group of syndromes characterized by severe headaches, typically associated with reversible segmental constriction of cerebral arteries, and it may be complicated by ischemic or hemorrhagic stroke (2). RCVS is the most important cause of thunderclap headache (3), commonly reversible, but several neurological complications including seizure, ischemic infarcts, and hemorrhage may happen.

## Pathophysiological Basis

Several pathophysiological mechanisms have been proposed for both syndromes but pathogenesis remains unclear (1, 2). The role of disordered cerebral vascularization, autoregulation, and endothelial function has been supposed but, due to their heterogeneous manifestations and pleiomorphic nature of the lesions, probably more than one mechanism is involved in etiology and they may vary in different clinical settings (1, 4).

In both syndromes, a blood flow dysregulation has been suggested to have a causative role but other mechanisms as immune system dysregulation or endothelium dysfunction may play a role in pathogenesis or in clinical course (1, 5). However, the occurrence of both syndromes in the same patients (6–10) makes conceivable a common origin or a common pathophysiological pattern making differential diagnosis difficult (11, 12), even if a possible overlap syndrome could not be completely ruled out (13).

### PRES

Pathophysiology of PRES remains controversial but the mechanism of a rapid increase in blood pressure is supposed to be central. Blood flow autoregulation indicates the capability of a tissue or a vascular bed to maintain a constant perfusion despite changes in systemic blood pressure (14, 15). Hypertension and associated conditions have often been indicated as key factors for the development of PRES and emergent pressure treatment was associated with symptoms relief in hours or days (16–18); however, also normo- or hypotensive patients with PRES have been described (19). Blood pressure rise and acute changing of blood pressure are commonly encountered in PRES; whether their role is causative or a secondary effect of the syndrome is still debated (4, 17, 20).

Some studies reported a possible immunological activation more than an effect of systemic hypertension (17, 21). Impaired cerebral autoregulation causing an increase in cerebral blood flow and endothelial dysfunction with cerebral hypoperfusion were indicated as possible mechanisms (4). Endothelial dysfunction may be the most relevant mechanism in preeclampsia or cytotoxic therapy (4, 20). Cytokines, lactate serum dehydrogenase (LDH), and vascular endothelial growth factor (VEGF) have been supposed to regulate vascular permeability (22) and endothelial dysfunction was reported in chronic renal failure, hemolytic uremic syndrome, and lupus nephritis (1, 5).

### RCVS

RCVS is more common in women than in men, and it has been described in patients aged from 10 to 76 years with a peak at around 42 years (2). Incidence is uncertain, but considering rates of patient recruitment into clinical series, RCVS does not appear rare. The first single center large series was reported in 2007 (23). Recent reports have proposed an increase in incidence of RCVS, but it is unclear whether this observation reflects a true increase in the incidence or an epiphenomenon due to physician awareness and diffusion and improved imaging techniques (3). Pathophysiology of RCVS remains unknown but a possible role of a transitory cerebral vascular autoregulation dysfunction and blood–brain barrier (BBB) breakdown have been postulated (24). A transitory spontaneous or provoked central vascular discharge may cause the alteration, explaining the reversible nature of RCVS and, because cerebral blood vessels are densely innervated with sensory afferents from trigeminal nerve, these mechanisms may contribute to the severe and acute headache (25).

## Clinical Features

### PRES

PRES may affect all age groups with patients ranging from 2 to 90 years (26) but commonly affects the young or middle-aged adults with a female predominance even after exclusion of patients with eclampsia (27–29). The incidence in pediatric population is low between 0.04 and 0.4% in pediatric intensive care units (30), whereas in adults, it is reported between 2 and 25% in patients after bone marrow transplantation, in about 10% of patients with autoimmune disease and in about 25% of patients with infection, sepsis, and shock. Also, end-stage renal disease may be a consistent risk factor (31–33).

PRES patients may show several neurological symptoms, commonly headache, impaired visual acuity, or visual field deficits, but confusion, focal neurological deficits, and disorders of consciousness with seizures may also occur. Clinical presentation has a great variability and course may depend on comorbidities and precipitating factors, but more than 90% of patients have typical clinical and neuroradiological features (34).

At the onset, neurological symptoms may be confusing and not specific with encephalopathy and seizures. Visual disturbance, hypertension, renal failure, and chemotherapy may be predicting factors for PRES (35) but diagnostic process may be challenging. Prognosis is generally favorable because in most patients both clinical symptoms and imaging lesions are reversible. On the other hand, long-term neurological impairments including epilepsy have been observed (16) and in-hospital death may involve one out of three patients with hemorrhagic PRES (36, 37).

PRES is usually monophasic and reversible (38) but recurrence has been reported (39).

### RCVS

Clinical setting of RCVS is quite different from PRES (Table 1). Conditions associated with RCVS are commonly pregnancy, even without eclampsia, neurosurgical procedure, and vasoactive drug use; RCVS typically involves women between the ages of 20 and 50. Clinical course is generally self-limiting but recurrences and complications till death may occur (23, 40). Unusual, recent, severe headaches of progressive or sudden onset, associated or not with focal neurological deficits and seizures, may be initial clinical scenario. Thunderclap headache is one of the chief clinical presentations defined as “any severe headache peaking within 1 min, and ‘non-thunderclap' headache any headache with a mild to severe intensity, peaking in more than 1 min” (24, 41). RCVS usually has a self-limiting course; resolution of symptoms happens by 3 weeks and resolution of vasoconstriction should occur by 3 months. A more rapidly progressive course of RCVS may lead to permanent disability or even in-hospital death in 5–10% of patients. Some factors such as glucocorticoid therapy, intra-arterial vasodilator therapy, and infarction on baseline imaging may be associated with poor outcome (42).

**Table 1:** Clinical and radiological features in PRES and RCVS patients.

	**PRES**	**RCVS**
**CLINICAL FEATURES**		
Associated clinical conditions	Immunosuppression, malignancy, pre-eclampsia, renal failure, dialysis, autoimmune disorders, infection, sepsis, hypertension, transplantation, chemotherapeutic medications, idiopathic	Pregnancy and puerperium, exposure to vasoactive drugs and blood products, head trauma, neurosurgical procedures, idiopathic
Headache	Moderate/severe	Thunderclap type
Seizures	Common	Uncommon
Encephalopathy	Common	Uncommon
Visual impairment	Common	Uncommon
Focal neurological deficits	Uncommon	Common in ischemic and hemorrhagic lesions
CSF analysis	Normal or near normal	Normal or near normal
**RADIOLOGICAL FEATURES**		
Useful MRI protocols	FLAIR, DWI, ADC, SWI, CE-MRA	FLAIR, DWI, ADC, SWI, CE-MRA
Usefulness of DSA	Rarely	Yes
Lesions distribution	Symmetric	Asymmetric
Edema distribution	*Common*: parieto-occipital pattern, holohemispheric watershed pattern, superior frontal sulcus pattern *Uncommon*: partial or asymmetric expression of above primary patterns	Uncommon: PRES-like
Ischemic lesion	Uncommon	Common
Hemorrhage lesion	*Common*: punctate type *Uncommon:* ICH, SAH	Common: SAH, ICH
Vasocostriction	Uncommon	Common: string-of-beads, distal vascular pruning
Contrast enhancement	Superficial leptomeningeal enhancement, gyral cortical enhancement	Uncommon

*PRES, posterior reversible encephalopathy syndrome; RCVS, reversible cerebral vasoconstriction syndrome; CSF, cerebrospinal fluid; FLAIR, fluid-attenuated inversion recovery; DWI, diffusion-weighted imaging; ADC, apparent diffusion coefficient; SWI, susceptibility-weighted imaging; CE-MRA, contrast enhancement magnetic resonance angiography; DSA, digital subtraction angiography; ICH, intracerebral hemorrhage; SAH, subarachnoid hemorrhage*.

## Role of Neuroimaging in Diagnosis

PRES and RCVS share some clinical and pathophysiological features and neuroimaging are mandatory in differentiating these syndromes. PRES at the onset is heterogeneous because of lesions distribution and features that occasionally resemble some RCVS features, suggesting an overlapping or a common pathway in their pathophysiological mechanisms (17, 43). On the other hand, radiological features, taken together with clinical context and symptoms may help in differential diagnosis. Conversely, RCVS patients, even if they show peculiar neuroradiological features such as hemorrhage and vasoconstriction pattern, may show features commonly observed in PRES (Table 1) such as vasogenic edema (23).

### PRES

In PRES, MRI shows a typical parieto-occipital pattern, but several patterns were described. Fluid-attenuated inversion recovery (FLAIR) sequences on MRI show almost symmetric hemispheric vasogenic edema involving subcortical white matter and overlying cortex, but other patterns were also found. Parietal–occipital regions may be involved in more than 90% of cases due to vascular cerebral dysregulation. Lesion distribution patterns include a holohemispheric watershed pattern, superior frontal sulcus pattern, a dominant parietal–occipital pattern, or partial or asymmetric expression of these primary patterns. These patterns may be useful to confirm the diagnosis, but notably type and severity of clinical presentation are associated neither with the pattern nor with the severity of brain edema (28).

Atypical presentations were reported in terms of regions involved (brainstem, spine, deep brain nuclei) or lesions type not related with vasogenic edema such as diffusion restriction, contrast enhancement, or hemorrhage (18, 44, 45).

**Figure 1:**
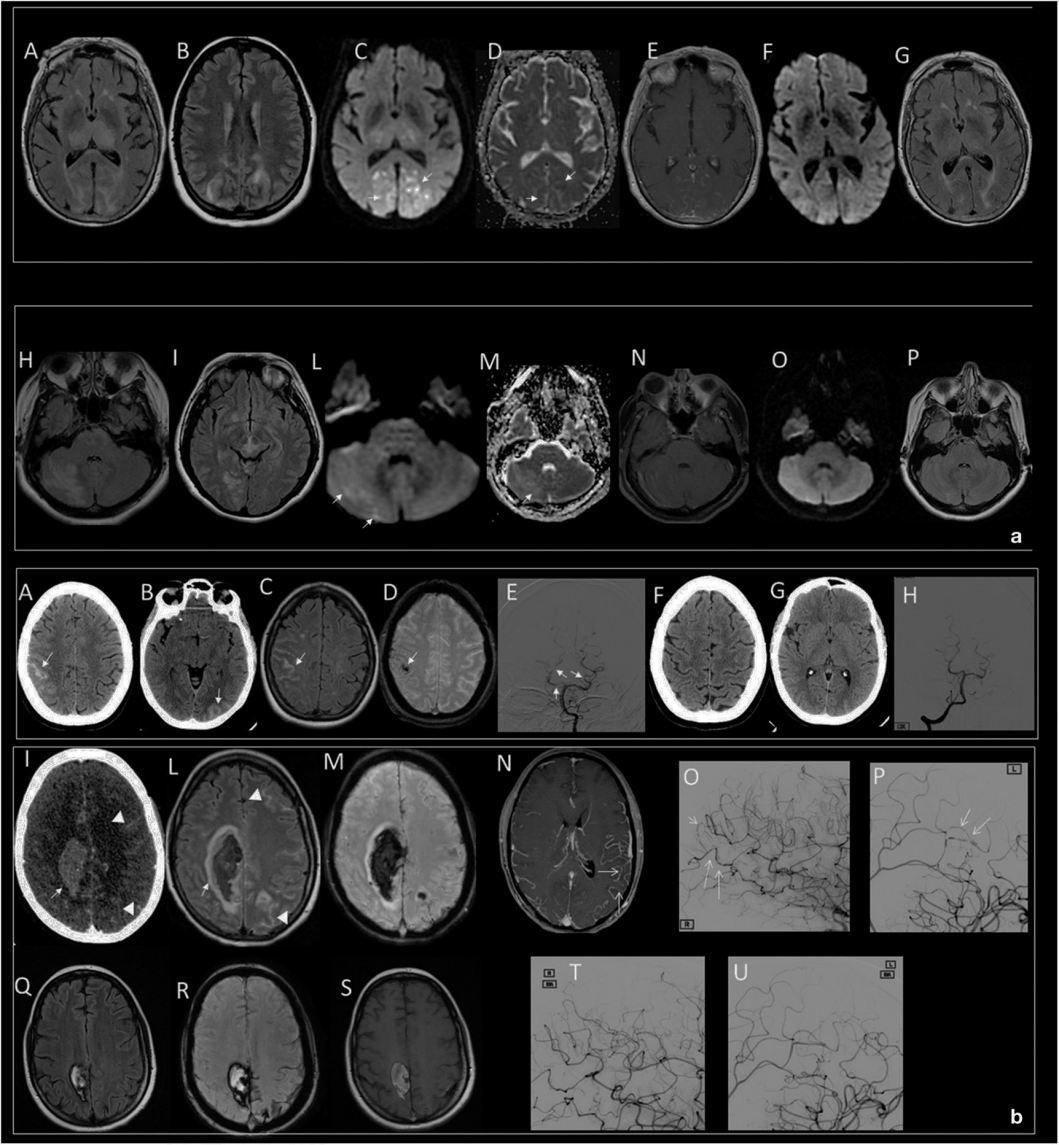
**(a)** Typical dominant parietal–occipital pattern in a patient with PRES at the onset (A–E) and after 15 days (F,G). (A,B,G) FLAIR MR images; (C,F) DWI MR images; (D) ADC map; (E) T1 C+ MR image. Edema involves the parietal and occipital cortex and white matter (A,B); small, patchy, or punctate hyperintensity in DWI (white arrows in C) corresponding to hypointensity in ADC map (white arrows in D) characterize the cytotoxic edema within diffuse vasogenic edema; gyral or leptomeningeal enhancement is shown in occipital regions (E). Note resolution of the lesions 15 days after the onset (F,G). Atypical involvement of the brainstem associated to occipital pattern in a patient with PRES at the onset (H–N) and after 18 days (O,P). (H,I,P) FLAIR MR images; (L,O) DWI MR image; (M) ADC map; (N) T1 C+ MR image. Edema involves the right cerebellum, brainstem, and occipital cortex and white matter (H,I); iso-intensity with punctate foci of hyperintensity in DWI (white arrows in L) and hyperintense signal in ADC characterizes the vasogenic edema (white arrow in M), no enhancement is shown (N). Note resolution of the lesions 18 days after the onset (O,P). **(b)** Intracranial subarachnoid hemorrhage in a patient with RCVS at the onset (A–E) and after 2 months (F–H). Axial CT (A,B) shows hyperdense subarachnoid hemorrhage in the right frontal (white arrow in A) and left occipital lobes (white arrow in B); axial FLAIR MR (C) confirms subarachnoid hemorrhage as hyperintense sulci (white arrow in C); SWI MR images (D) show a component of subarachnoid hemorrhage as hypointense focus within a frontal sulcus (white arrow in D); catheter angiography of vertebro-basilar arteries demonstrate vessel irregularities with focal vasoconstriction (white arrow in E). Note resolution of SAH (F,G) and vessel irregularities (H) after 2 months. Intraparenchymal hematoma and subarachnoid hemorrhage in a patient with post-partum RCVS at the onset (I–P) and after 3 months (Q–U). Axial CT (I) shows hyperdense parenchymal hematoma in the right frontal lobe (white arrow in I) and subarachnoid hemorrhage in the left frontal lobe (white head of arrows in I); FLAIR MR (L) shows vasogenic edema marginally at the right parenchymal hematoma (white arrow in L) and left subarachnoid hemorrhage as hyperintense sulci (white head of arrows in L); SWI MR image (M) shows the hypointense signal of the parenchymal and subarachnoid hemorrhage due to acute phase of hemorrhage; contrast-enhanced MRA (CE-MRA) (N) images show vasoconstriction of some distal branches of middle cerebral arteries (with arrows in N); catheter angiography of internal carotids confirms diffuse irregularities with multifocal narrowings throughout the cerebral vasculature with a “string-of-beads” appearance (white arrows in O,P). Note reduction of ICH and SAH (Q–S) and disappearance of multifocal narrowings of distal branches of middle cerebral arteries after 3 months (T,U).

MRI by FLAIR, diffusion-weighted imaging (DWI), and apparent diffusion coefficient (ADC) are useful in differentiating types of edema in PRES. Usually, the vasogenic nature of edema is a hallmark of PRES even if small areas of cytotoxic edema may occur. Iso-intense or hyperintense signal on DWI and hyperintense signal on ADC mapping are typical appearances of vasogenic edema whereas hyperintense signal on the DWI and hypointense signal in the ADC are a hallmark of cytotoxic edema (46). Regions of reduced diffusion usually are small, punctate, or patchy and are shown within confluent lesions of vasogenic edema (Figure 1a); extensive regions of reduced diffusion are rarely described (43). Vasogenic edema can generally be completely reversible but reduced ADC values are not a sign of irreversibility (46).

In PRES patients, on post-contrast T1WI MRI, a superficial leptomeningeal enhancement is the most common pattern but a nodular and, in about one-third of patients, a combined leptomeningeal (36, 44) and gyral cortical enhancement can be observed (47).

Several patterns of hemorrhage have been described, such as large hematomas with mass effect, subarachnoid hemorrhage (SAH) or multiple minute foci and microhemorrhages, but the most common is the punctate type (37, 47). Intracranial hemorrhage is encountered in PRES patients with an incidence of ~15% (4). Some patients with PRES may show some RCVS-like features such as cerebral vasoconstriction (2).

### RCVS

On MRI, bilateral symmetric parieto-occipital lesions, typical for PRES, are not characteristic for the RCVS. However, PRES-like reversible cerebral edema have been reported in 17–38% of patients with RCVS, suggesting common origins or mechanisms for both conditions (43, 47, 48).

The classical radiological presentation assessed by MRA or conventional angiography includes cerebral vasoconstriction, with at least two narrowings in the same artery, on two different cerebral arteries; commonly, arterial abnormalities disappear in <3 months (23). About one-third of patients develop brain hemorrhage or ischemic strokes, or reversible brain edema. SAH or intraparenchymal hemorrhage are common complications of RCVS (18, 37).

Hemorrhage is typically isolated SAH occasionally associated with superficial intracerebral hemorrhage (ICH) (Figure 1b). Rarely, isolated deep ICH may occur, making differential diagnosis difficult (48). Several factors have been associated with hemorrhage in RCVS such as migraine history and female gender, but despite the dramatic onset, over 90% of patients have excellent clinical outcome (42).

Catheter angiography is the gold standard for diagnosis, and MR angiography (MRA) and CT angiography (CTA) may disclose vessel irregularities with diffuse or focal vasoconstriction (Figure 1b), vasodilation, or a “string-of-beads” appearance; moreover, reversible distal vascular pruning may also be revealed (49).

Wall enhancement has been used in differential diagnosis between RCVS and vasculitis because it has been described as a marker of vasculitis but results of several studies are controversial and utility in differential diagnosis is debated (50, 51).

## Role of Neuroimaging in Understanding Pathophysiology

Common features observed in PRES and RCVS make conceivable a shared pathophysiological pathway or common effects on the intracranial vascularization (12).

### PRES

Systemic hypertension with tissue hyper-perfusion due to failed autoregulation was a popular theory (1, 52), even if an alternative theory of vasoconstriction, reduced perfusion, and ischemia may explain most of the lesions and edema localization in PRES (17, 20). The findings of post-contrast T1WI MRI, showing a superficial leptomeningeal enhancement in about one-third of patients (38), gyral cortical enhancement (49), and microhemorrhages detected by susceptibility-weighted imaging (SWI) seem to confirm the abovementioned mechanisms followed by the breakdown of the BBB.

### RCVS

A possible pathophysiological role of BBB breakdown along with sympathetic overactivity and dysregulation of vascular tone was postulated (2, 3). A disturbance in cerebral vascular tone or in its control seems to be a critical element in RCVS. Vascular tone dysfunction may be spontaneous or caused by various exogenous or endogenous factors such as vasoactive drugs, tumors, endocrine factors, direct or neurosurgical trauma, and uncontrolled hypertension (2). Interestingly, Lee et al. confirmed BBB breakdown by contrast-enhanced fluid-attenuated inversion recovery (CE-FLAIR) on MRI performed within 7 days from clinical onset (53).

## Neuroimaging And Temporal Evolution

### PRES

Time course of the lesions have not been prospectively evaluated and only few case series reported very early examinations (54). After acute phase, most PRES patients show a complete recovery and long-term prognosis is generally good but persistent neurological impairments and death may be noted in about 3–6% of patients (4). Fatalities may reach 30% of patients in hemorrhagic (18) or in malignant PRES (55). Neuroradiologic criteria for malignant PRES are edema with associated mass effect, brain hemorrhage exerting mass effect, effacement of basal cisterns, transtentorial, tonsillar, or uncal herniation (55).

### RCVS

In RCVS, symptoms typically follow a self-limiting, monophasic course, with resolution by 3 weeks (56, 57) but resolution of vasoconstriction may take 3 months (2). Outcome for most patients is good; however, some patients have a delayed clinical worsening in the first few weeks often due to the development of extensive ischemic or hemorrhagic infarcts. Extensive hemorrhagic lesions need a closer attention due to a possible mass effect. A fulminant course of RCVS leading to permanent disability or death can been countered in 5–10% of patients (25). RCVS encountered in the postpartum period (58) warrants a particular care because it may have a fulminant course, with multifocal infarct or intracranial hemorrhage and extensive vasogenic edema (57, 59). Sequential examination by MRI and CT are warranted to catch initial worsening signs.

## Role of Neuroimaging in Prognosis

Prognosis is commonly good for both syndromes, but some patients may show neurological sequalae or even death (16, 57); then, neurological worsening could not indicate an alternative diagnosis. Often, in these cases, central nervous system vasculitis has been taken into account in the differential diagnostic pathway, adding further unnecessary and invasive tests or therapies (2) such as potent chemotherapeutic agents with potentially serious adverse effects (56). A previous study reported a post-angiogram worsening in RCVS (23) but a similar proportion of cases of clinical worsening within 24 h after MRA or CTA was reported (57), indicating a natural course of disease rather than a side effect of catheter angiography.

### PRES

Reversibility of the lesions is a hallmark of PRES, but occasionally a mismatch between radiological reversibility and good prognosis may be noted. Most patients have a reversion of imaging abnormalities, but permanent tissue damages were also observed (16); on the other hand, some patients show radiological reversibility, but poor outcome (5), mainly due to comorbidities and complications (60). In PRES, acute hypertension is a common observation, but it is not related with either poor prognosis (54) or hemorrhage rate or type (16, 18). Unfavorable outcome is often associated with chemotherapy and sepsis, but notably, these patients have serious underlying medical conditions (16, 54).

In patients with PRES brainstem involvement, an early evidence of hemorrhage and other MR patterns as massive edema were associated with poor prognosis (54).

High DWI signal intensity and low or normal ADC mapping values are associated with cerebral infarction (54). Consequently, DWI and ADC mapping may help in predicting conversion to infarction and then tissue damage (61).

The association between contrast-enhancement (CE) pattern and prognosis in PRES is still debated (44), but recent studies reported a link among poor outcome, hemorrhage, and cytotoxic edema (54, 62). Contrast enhancement shows the breakdown or an augmented permeability of the BBB (63), but being a temporal phenomenon, it could be transitory, suggesting different stages in the integrity of the BBB (44).

### RCVS

About 25% of RCVS patients develop complications, including cortical subarachnoid hemorrhage (cSAH), convulsions, and ischemic events (25) secondary to arterial vasoconstriction and cerebral edema (64). In a recent review about fatal causes of RCVS, a good prognosis was found in 78–90% of patients with RCVS, but a mortality rate of 1–5% mainly occurred in postpartum and pregnancy. Fatal course was linked also to initial focal signs on neurological examination, rapid clinical decline, or initial abnormal imaging suggestive of stroke (64).

## RCVS Associated With PRES

These two clinical conditions were reported in same patients (6–10) and some revisions were reported in about 10% of RCVS patients' symmetrical high-intensity lesions in posterior zones of the brain as observed in PRES patients (56, 64). These observations make conceivable a common origin or a common pathophysiological pathway but due to the lack of prospective studies, neither overlapping syndrome nor a temporal phenomenon could be ruled out (13, 65). It is probable that BBB breakdown is a dynamic process or a continuum in which either microvascular damage due to endothelium dysfunction or vascular autoregulation or both may trigger the process dependent on the patient's risk factors (toxic or pressure's changes); this cascade of events may lead to either PRES or RCVS or both.

## Possible Developments in Neuroimaging

Possible research fields in which neuroimaging may develop could involve understanding of the pathophysiology and forecasting prognosis. In particular, the role of BBB and vascular autoregulation should be investigated. Recent researches investigated a possible role of BBB breakdown in RCVS (53). Serial MRIs in the first hours after symptoms onset may give new insight into understanding the pathophysiology of both syndromes. Moreover, early neuroradiological heraldic signs suggestive of malignant PRES or extensive ICH in RCVS are lacking. New research are mandatory in discovering these early signs that could have a significant impact in patient management. Early discovery of patients at highest risk for deterioration may be helpful to assess an appropriate triage and a consequent level of care and monitoring (57), particularly in high-risk patients such as postpartum RCVS with intracranial hemorrhage (66).

## Conclusion

Pathophysiological mechanisms of PRES and RCVS are still unknown. Whether PRES and RCVS are independent syndromes and sometimes overlapped or part of a continuum process, these theories are still debated. However, some common characteristics make conceivable a common origin somehow linked with cerebral autoregulation, endothelial dysfunction, and BBB breakdown.

The developing and spreading of MRI and prospective neuroradiological studies at a very early time from clinical onset, linked with increased clinical awareness, may help in the diagnosis, thus enhancing recognition and avoiding unnecessary or dangerous treatments. Moreover, neuroimaging may give new insights into understanding etiologies and discovering pathophysiologic processes and, in more severe cases, it may help in personalizing treatment and thus improving outcome.

## Author Contributions

FP wrote the majority of the manuscript. RC wrote portions of the manuscript and supplied case material and research on the topic. MD provided feedback in the manuscript preparation and research on the topic.

### Conflict of Interest

The authors declare that the research was conducted in the absence of any commercial or financial relationships that could be construed as a potential conflict of interest.
